# Development and validation of a prognostic nomogram for ambulatory patients with advanced cancer

**DOI:** 10.1002/cam4.1582

**Published:** 2018-06-01

**Authors:** Carlos Eduardo Paiva, Bianca Sakamoto Ribeiro Paiva, Naitielle de Paula Pântano, Daniel D’Almeida Preto, Cleyton Zanardo de Oliveira, Sriram Yennurajalingam, David Hui, Eduardo Bruera

**Affiliations:** ^1^ Department of Clinical Oncology Barretos Cancer Hospital Barretos SP Brazil; ^2^ Palliative Care and Quality of Life Research Group Post‐Graduate Program Barretos Cancer Hospital Barretos SP Brazil; ^3^ Researcher Support Center Learning and Research Institute Barretos Cancer Hospital Barretos SP Brazil; ^4^ Education and Research BP ‐ A Beneficência Portuguesa de São Paulo São Paulo SP Brazil; ^5^ Department of Palliative Care and Rehabilitation Medicine M.D. Anderson Cancer Center The University of Texas Houston TX USA

**Keywords:** development, neoplasias, prognostic, survival, validation

## Abstract

Predicting survival of advanced cancer patients (ACPs) is a difficult task. We aimed at developing and testing a new prognostic tool in ACPs when they were first referred to palliative care (PC). A total of 497 patients were analyzed in this study (development sample, n = 221; validation sample, n = 276). From 35 initial putative prognostic variables, 14 of them were selected for multivariable Cox regression analyses; the most accurate final model was identified by backward variable elimination. Parameters were built into a nomogram to estimate the probability of patient survival at 30, 90, and 180 days. Calibration and discrimination properties of the Barretos Prognostic Nomogram (BPN) were evaluated in the validation phase of the study. The BPN was composed of 5 parameters: sex, presence of distant metastasis, Karnofsky Performance Status (KPS), white blood cell (WBC) count, and serum albumin concentration. The C‐index was 0.71. The values of the area under the curve (AUC) of the receiver operating characteristic (ROC) curve were 0.84, 0.74, and 0.74 at 30, 90, and 180 days, respectively. There were good calibration results according to the Hosmer‐Lemeshow test. The median survival times were 313, 129, and 37 days for the BPN scores <25th percentile (<125), 25th to 75th percentile (125‐175), and >75th percentile (>175), respectively (*P* < .001). The BPN is a new prognostic tool with adequate calibration and discrimination properties. It is now available to assist oncologists and palliative care physicians in estimating the survival of adult patients with advanced solid tumors.

## 1 BACKGROUND

Although palliative care (PC) previously focused on end‐of‐life care, there is an increasing understanding that PC must be integrated earlier into the continuum of cancer care in conjunction with other therapies that are intended to prolong life, such as chemotherapy.^1^ Early PC has been shown to improve health‐related quality of life (HRQOL), mood, and symptom scores.^2^, ^3^, ^4^ Additionally, early PC may increase patient satisfaction with care^5^ and facilitate the optimal and appropriate administration of anticancer therapy, especially during the final months of life.^2^

Oncologists frequently face the difficult task of estimating prognosis in patients with incurable malignancies. Their prediction of prognosis informs decision‐making ranging from recommendations of cancer treatments to hospice enrollment.^6^ Their subjective judgment for predicting survival is often inaccurate and usually too optimistic, which may result in overly aggressive cancer treatment. Actuarial judgment, based on the assessment of statistically derived key factors, has the potential to improve prognostic accuracy.^7^, ^8^

The Karnofsky Performance Status (KPS),^9^ the Palliative Performance Scale (PPS),^10^ and the Eastern Cooperative Oncology Group Performance Status (ECOG‐PS)^11^ have been developed to measure the functional status of patients with cancer and are the most widely used functional scales with prognostic potential.

Prognostic models have been developed and validated, but to date, none are able to provide accurate estimates over the spectrum of advanced illness.^6^ Some classical prognostic factors in oncology, such as tumor staging, histologic grade, and genetic factors, do not appear to have a prognostic impact on advanced cancer patients in PC.^6^, ^12^ Thus, other prognostic markers have been investigated in this clinical situation.

There is a lack of studies that evaluated the prognosis of outpatients with advanced cancer who are receiving antineoplastic treatment and concomitantly undergoing PC. Most of the studies describe a sample of patients with advanced disease, a low functional performance, and a short life expectancy.^12^ Considering that providing early PC improves HRQOL and symptom management, this study aims at developing and validating the Barretos Prognostic Nomogram (BPN), in terms of calibration and discrimination, for the prediction of survival of outpatients with advanced cancer when first referred to PC.

## 2 METHODS

### 2.1 Study design

This prospective, observational study was conducted at a cancer hospital in Barretos‐SP, Brazil. This study encompassed 2 distinct phases: (1) development (from March 2011 to April 2012) and (2) validation (from April 2014 to October 2014). All the included patients were followed until death or the end of the study.

### 2.2 Study sample

In the development phase, patients were included if they had incurable cancer and attended the Palliative Care Outpatient Clinic for their first consultation, regardless of whether or not they were undergoing palliative antineoplastic treatment. In the validation phase, patients were included if they had solid advanced cancer (ie, incurable metastatic or locally advanced disease) and were referred by the clinical oncology team for the first time to the Palliative Care Outpatient Clinic. In both phases, patients were excluded if they were <18 years of age, had any cognitive or psychiatric diseases that rendered them incapable of answering the questionnaire items, or refused to participate. All the patients were included by convenience.

### 2.3 Data collection

#### 2.3.1 Development sample

Data regarding patient characteristics, KPS, HRQOL indices, cancer symptoms, and blood samples were collected in the initial evaluation by research nurses. Body mass index (BMI) and the physical examination of edema and ascites were performed by a nutritionist.

Health‐related quality of life was measured by the 30‐item questionnaire of the European Organization for Research and Treatment of Cancer (EORTC) QLQ‐C30,^13^ and symptoms were measured by the Edmonton Symptom Assessment System (ESAS).^14^ Only the variables from the QLQ‐C30 and the ESAS having biologic background justifying a prognostic role were included in the univariate survival analysis (Table S1).

#### 2.3.2 Validation sample

Data regarding patient characteristics, KPS, and blood samples (complete blood count and serum albumin levels) were collected by the research nurses during the initial evaluation.

### 2.4 Data analysis

Patients were followed by telephone interviews every 15‐30 days until death. Follow‐up was terminated after reaching a predefined rate of at least 70% of deaths, that is, 155 events from 221 patients in the development phase and 194 events from 276 patients in the validation phase. Overall survival (OS) times were calculated from the time of study inclusion until death for any reason.

In the development phase, statistical analyses were performed by univariate and multivariate Cox regression analyses. All variables with *P*‐values <.2 were entered in the multivariate model. A stepwise backward method was used for the selection of the variables. The final prognostic model was used to develop the BPN.

In the validation sample, measures of discrimination and calibration were obtained.

#### 2.4.1 Discrimination

The discrimination properties were evaluated by means of survival analysis, receiver operating characteristic (ROC) curve calculation, concordance index (C‐index) and Kolmogorov‐Smirnov (K‐S) goodness of fit.

The BPN scores were used as continuous variables and the occurrence of death (yes/no) as a categorical variable in ROC curve analyses to identify the points of the highest prognostic accuracy. Sensitivity, specificity, and positive and negative predictive values were calculated to predict the risk of death at 30, 90, and 180 days.

The K‐S goodness‐of‐fit test was used to measure the ability of prognostic tools to discriminate between groups (alive vs death). This test statistic should be compared to a tabulated value according to the sample size. Thus, a value higher than 0.081 was expected (α** **=** **5%, n** **=** **276; 1.36/√n).^15^

For the C‐index, a value of 0.5 indicates that there is no discrimination, whereas a value of 1.0 indicates perfect discrimination between the expected and the observed outcomes.^16^

#### 2.4.2 Calibration

The Hosmer‐Lemeshow goodness‐of‐fit test was used to evaluate the calibration of the nomogram. It evaluates the quality of fit of the model (how the observed results fit those predicted by the model), and adequate results should be nonstatistically significant (*P* > .05).^17^

Statistical analysis was performed using Statistical Package for the Social Sciences (SPSS) software (version 19.0) and R statistical software. The BPN was constructed using the nomogram function (rms package version 4.0) and the coxph function (Survival package version 2.37‐4) of R statistical software version 2.15.1. *P*‐values <.05 were considered statistically significant.

#### 2.4.3 Sample size estimation

The sample size of the development phase was estimated considering a ratio of at least 1:10 between the number of events vs the number of predictors in the multivariate model. Therefore, a model with 15 predictors would require a minimum sample size of 150 events. Follow‐up was terminated after reaching a predefined rate of at least 70% of deaths (ie, 155 events from 221 patients). The sample size of the validation set was calculated considering a proportion of correct answers of 80% (true positives and true negatives), an absolute error of 5%, and a level of significance of 5%. The sample size calculated was 246 patients.^18^ Considering a 10% rate of lack of information, the decision was made to include a minimum of 270 patients for the present phase. Taking into account that a subgroup of advanced cancer patients may present longer survival times than expected (years of survival), due to logistic research issues, the follow‐up in the validation cohort was also planned to stop after reaching at least 70% of deaths.

## 3 RESULTS

A total of 497 patients were analyzed in this study; 221 were included in the training and 276 in the validation sets. The median ages were similar in the 2 cohorts (development median** **=** **61 years; validation median** **=** **60.2 years). There were fewer patients receiving antineoplastic therapies in the development sample in comparison with the validation sample (47.5% vs 67.8%, respectively). On the other hand, patients in the development sample had higher median KPS scores than those in the validation sample (development KPS** **=** **80; validation KPS** **=** **60). In both the development and validation cohorts, the most common primary tumors were breast (16.7% and 23.6%, respectively) and upper digestive (17.6% and 14.5%, respectively; Table 1). The median OS (95% confidence interval [CI]) times of the development and validation cohorts were 166 (135‐197) days and 124 (104‐144) days, respectively.

**Table 1:** Characteristics of patients in the training and validation cohorts

Characteristics	Training set (n** **=** **221)		Validation set (n** **=** **276)	
	N	%	N	%
Age				
Median (p25‐p75; years)	61.0	52‐70.5	60.2	52.6‐69.4
<60 y	101	45.7	140	50.7
60‐74 y	89	40.3	100	36.2
≥75 y	31	14.0	36	13.0
Gender				
Woman	109	49.3	164	59.4
Man	112	50.7	112	40.6
Work				
Active	26	11.8	68	24.6
Inactive	192	86.9	207	75.0
Missing	3	1.4	1	0.4
Site of metastasis				
Lung (yes)	59	26.7	92	33.3
Hepatic (yes)	41	18.6	93	33.7
Bone (yes)	62	28.1	102	37.0
CNS (yes)	15	6.8	26	9.4
Treatment				
Antineoplastic	105	47.5	187	67.8
PC only	116	52.5	89	32.2
KPS				
Median (p25‐p75; score)	80	60‐90	60	50‐70
0‐50 score	23	10.4	29	10.5
60‐70 score	80	36.2	141	51.1
80‐100 score	117	52.9	29	10.5
Missing score	1	0.5	0	0
Primary tumor site				
Breast	37	16.7	65	23.6
UGI	39	17.6	40	14.5
HN	18	8.1	23	8.3
LGI	24	10.9	36	13.0
Lung	29	13.1	39	14.1
Urological	30	13.6	25	9.1
Gynecological	22	10.0	27	9.8
Hematologic	6	2.7	0	0
Skin and soft tissue	11	5	10	3.6
Unknown primary	5	2.3	8	2.9
CNS	0	0	3	1.1

CNS, central nervous system; HN, head and neck; KPS, Karnofsky performance status; LGI, lower gastrointestinal; p25, 25th percentile; p75, 75th percentile; PC, palliative care; UGI, upper gastrointestinal.

### 3.1 Construction of the prognostic nomogram

**Figure 1:**
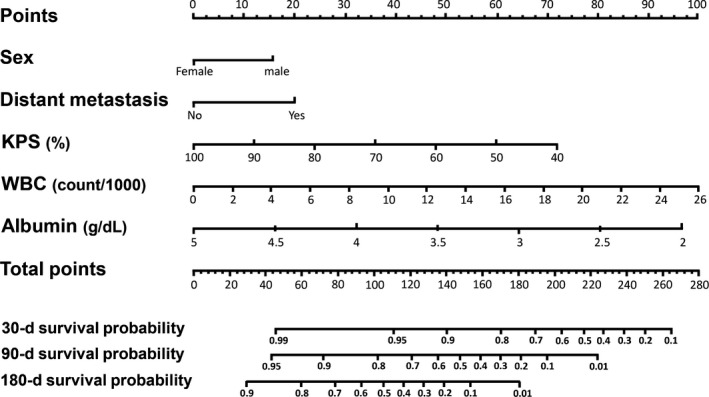
The Barretos Prognostic Nomogram (BPN). Points are assigned for sex, presence of distant metastasis, Karnofsky Performance Status (KPS), white blood cell (WBC) count, and serum albumin concentration by drawing a line upward from the score values to the “points” line. The summed points (5 variables) are plotted on the “total points” line. The “total points” line yields prediction of 30‐, 90‐, and 180‐d survival by drawing a line downward

Thirty‐five variables with potential prognostic capacity were individually evaluated using univariate Cox regression analyses (Table S1). Patient‐related variables (age, sex, performance status), tumor‐related variables (type of cancer, type of treatment, site of metastasis), nutritional variables (BMI, feeding tubes, ascites, peripheral edema), and several laboratory examinations (CRP, albumin, LDH, calcium, hemoglobin, WBC, lymphocytes, monocytes, platelets) were included in the analyses. Additionally, several cancer symptoms and HRQOL indices were analyzed. Only variables with *P*‐values <.2 were included in the statistical models. Statistical models with and without the inclusion of cancer symptoms and HRQOL indices were created. However, the authors decided not to include variables extracted from questionnaires in the final prognostic model, as the requirement to fill in questionnaires would make it difficult to use the tool in clinical practice. Lung metastasis, liver metastasis, and “any metastasis” were strongly correlated with each other; thus, only “any metastasis” was included in the multivariable model. After a backward stepwise method, 5 variables were maintained in the final model (Table 2, Table S2). The nomogram is depicted in Figure 1. How to use the nomogram is described in the Figure S1. The same 5 prognostic predictors were retained in the final Cox regression model when patients with hematologic cancers (n** **=** **6) were excluded from the analyses (Table S3).

**Table 2:** Final Cox proportional hazards regression model for multivariate analysis

Variables	B (SE)	Exp (B)	95% CI	*P*‐value
Female	−0.373 (0.176)	0.689	0.488‐0.972	.034
KPS	−0.030 (0.006)	0.971	0.959‐0.982	<.001
Albumin	−0.966 (0.162)	0.380	0.277‐0.522	<.001
Distant metastasis	0.587 (0.208)	1.799	1.196‐2.706	.005
WBC count	0.086 (0.023)	1.089	1.042‐1.139	<.001

CI, confidence interval; KPS, Karnofsky Performance Status; SE, standard error; WBC, white blood cell.

### 3.2 Validation of the nomogram

The average score for the BPN was 151.4 ± 42.1 points (mean ± SD). This score represents the following survival probabilities in the nomogram: 30 days (87%), 90 days (45%), 180 days (10%), and 360 days (1%).

Each of the 5 nomogram parameters produces an individual score; the sum of these scores was used for statistical analysis. The cutoff points for 30, 90, and 180 days were 162, 150, and 142, respectively. The highest value of the area under the curve (AUC) for the ROC curve was obtained at 30 days (AUC** **=** **0.84). Likewise, the highest values of sensitivity (78.43%) and specificity (74.88%) were also identified at 30 days (Table 3). The K‐S results from the 30‐, 90‐, and 180‐day evaluations were all higher than the predefined value of 0.08. The C‐index value was 0.71. Regarding the Hosmer‐Lemeshow test, the findings for 30, 90, and 180 days were all considered adequate (*P* > .05; Table 3).

**Table 3:** Calibration and discrimination results of the Barretos Prognostic Nomogram

Characteristics	30 d	90 d	180 d
Cutoff scores	162	150	142
Area under the ROC curve	0.840	0.743	0.741
Sensitivity	78.4%	66.3%	66.7%
Specificity	74.9%	65.2%	69.4%
NPV	93.6%	75.3%	55.7%
PPV	42.5%	54.7%	78.3%
K‐S	0.537	0.342	0.383
C‐index	0.71^a^	0.71^a^	0.71^a^
Hosmer‐Lemeshow	*P* ** **=** **.538	*P* ** **=** **.580	*P* ** **=** **.756

K‐S, Kolmogorov‐Smirnov; NPV, negative predictive value; PPV, positive predictive value; ROC, receiver operating characteristic.

a C‐index is a general measure that is not specified per moment.

**Figure 2:**
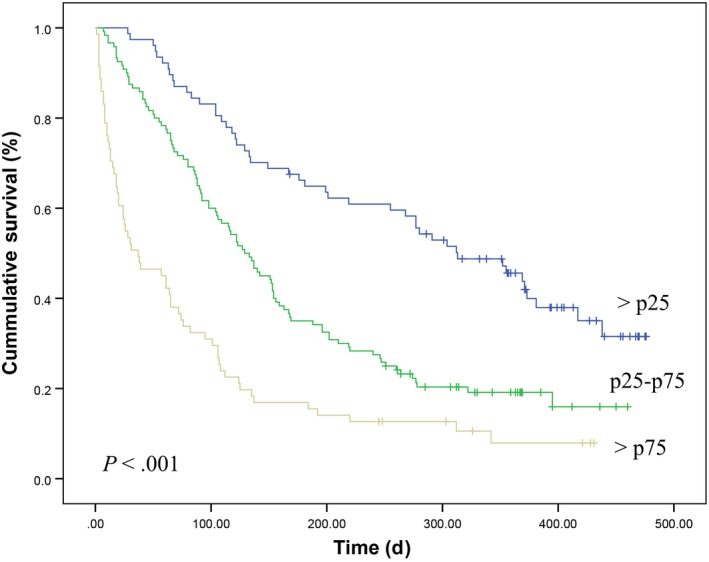
Overall survival curves according to Barretos Prognostic Nomogram (BPN) scores in validation sample. The 25th percentile (p25) represented a BPN total score of 125 and the 75th percentile (p75) a total score of 175

The Kaplan‐Meyer survival analysis was performed regarding the percentiles of the total score presented by the participants; that is, the 25th percentile (p25) represented a BPN total score of 125 and the 75th percentile (p75) a total score of 175. The following survival rates (%) were observed for 30, 90, and 180 days, respectively: scores <p25 (97.4%, 83.1%, 66.2%); scores p25‐p75 (87.5%, 64.2%, 35.1%); and scores >p75 (52.1%, 32.5%, 16.9%). The median survival (95% CI) times were 313 (225‐400) days, 129 (105‐152) days, and 37 (7‐66) days for the <p25, p25‐75, and >p75 percentile ranges, respectively (*P* ****<** **.001, Figure 2). The hazard ratios (HR) and 95% CIs were 1.0 (reference) for <p25; HR** **=** **2.045 (95% CI** **=** **1.436‐2.912) for p25‐p75; and HR** **=** **4.172 (95% CI** **=** **2.839‐6.130) for >p75 (*P* ****<** **.001).

## 4 DISCUSSION

In the present study, a new prognostic tool was developed that is designed to estimate the prognosis of ambulatory patients with advanced cancer. Considering the current recommendation of early integration of PC in oncology care,^1^ the prognostication of patients with “months” of survival needs to be better evaluated. A better prognostication of these patients can potentially help oncologists in defining the point of stopping/maintaining chemotherapy and hospice enrollment. Although the BPN should be considered a valid tool (with good calibration and discrimination properties), it seems to be more accurate in the end‐of‐life period but not earlier as originally planned.

Currently, several prognostic tools have been developed and externally validated. The majority of them were originally developed to estimate the prognosis of patients with days to weeks of survival.^19^, ^20^, ^21^, ^22^, ^23^, ^24^ To the best of our knowledge, no other prognostic tool has been designed to target outpatients when they are first referred to PC. From the available prognostic tools, the modified Glasgow Prognostic Score (mGPS),^25^ phase angle,^26^ and performance status scales 27 were externally validated in populations of cancer patients with median survival times longer than 2‐3 months. A recently published systematic review identified 7 different prognostic tools that were tested in patients with incurable cancers in different care settings and with different primary cancer types.^12^ The authors concluded that the PPS, the Palliative Prognostic Score (PaP), the Palliative Prognostic Index (PPI), and the GPS were all externally validated in large samples and predicted survival adequately. Recently, the PRONOPALL prognostic tool was externally validated in sample of 302 adult patients with incurable cancers; of those patients, there were 130 outpatients still receiving antineoplastic treatments with a median survival of 301 days.^28^

Among the 5 parameters of our nomogram, only KPS could be subjectively measured, as the interobserver reliability is not high among clinicians.^29^ Gender and presence of distant metastasis are both easily and objectively obtained, and the other 2 parameters are objectively measured from blood samples (white blood cell [WBC] count and serum albumin concentration). Two of the most commonly used prognostic tools, PPI and PaP, have many subjective parameters. From the 6 parameters of PaP, 4 are subjective; from the 5 parameters of PPI, all of them can be considered subjective. The PaP accuracy, for instance, has been improved when the clinician’s prediction of survival, a well‐known subjective measure, is excluded from the composite score.^30^

The BPN was tested regarding calibration and discrimination. Although it can be considered well validated for clinical use, the C‐index of 0.71 and the AUCs at 90 days (0.74) and 180 days (0.74) are not ideal. Four prognostic tools were compared in a multicenter prospective study conducted by Maltoni et al.^20^ The most accurate tools were PaP and Delirium‐PaP, with C‐indexes of 0.72‐0.73, which are quite similar to our findings. A Canadian study compared 3 classical performance scales in patients with advanced cancer and identified that the C‐indexes ranged from 0.63 to 0.64.^27^ In terms of C‐index, other prognostic tools have achieved better results. However, several other features need to be considered when choosing the best prognostic tool. Among other factors, ease of use (eg, the time to calculate the score and the need for blood collection) and the type of results available (survival probability or median survival time) are important.^31^ In this sense, most of the available tools determine prognostic categories with expected survival times. Although prognostic nomograms are commonly developed in general oncology, they are rarely used in PC. Like the BPN, a Spanish group 32 also developed a nomogram for the evaluation of patients with advanced cancer. With good calibration and a C‐index of 0.70 (similar to our results), the authors concluded that the Spanish nomogram was highly accurate.

### 4.1 Study limitations

The present study has several limitations. In the development sample, patients with hematologic cancers were included. However, the presence of distant metastasis was one of the clinical parameters included in the nomogram. Consequently, in the validation sample, no hematologic patients were included. Thus, BPN is not valid for use in patients with hematologic cancers. Considering that the development sample was composed of patients with good performance statuses (median KPS** **=** **80), the end of the nomogram reaches a minimum KPS of 40%. Thus, ambulatory patients who occasionally present with a KPS <40% are not well evaluated with the BPN. Another limitation is the fact that we included in the development phase patients already under evaluation by the palliative care team. Anyway, these patients were in their first consultation in the PC unit. Moreover, the development sample had a median overall survival of 5.5 months, which can probably be considered as an early PC referral.

### 4.2 Future perspectives

Future prospective studies are needed to compare the BPN with the other prognostic tools in patients with advanced cancer who are starting PC. Additionally, further analysis can test the prognostic accuracies of the BPN and other prognostic tools used sequentially and in different clinical settings.

## 5 CONCLUSION

The BPN is a new prognostic tool with adequate calibration and discrimination properties. Although it should be considered a valid tool to be used in the prognostication of adult patients with advanced solid tumors, its prognostic capacity is not ideal. Further strategies of prognostication and improvements in the BPN should be tested in future studies.

## CONFLICT OF INTEREST

No potential conflict of interest.

## ETHICS

All participants received the full information regarding the study and signed a consent‐to‐participate form prior to enrollment. Both phases had approval from the Ethics Committee of the BCH (development phase: HCB433/2011 and validation phase: HCB783/2014).
