# Supplementary figures and images for: Development and validation of a prognostic nomogram for ambulatory patients with advanced cancer
Source: Cancer Med. 2018 Jun 1;7(7):3003–10. doi: 10.1002/cam4.1582 (PMC6051167; doi:10.1002/cam4.1582)

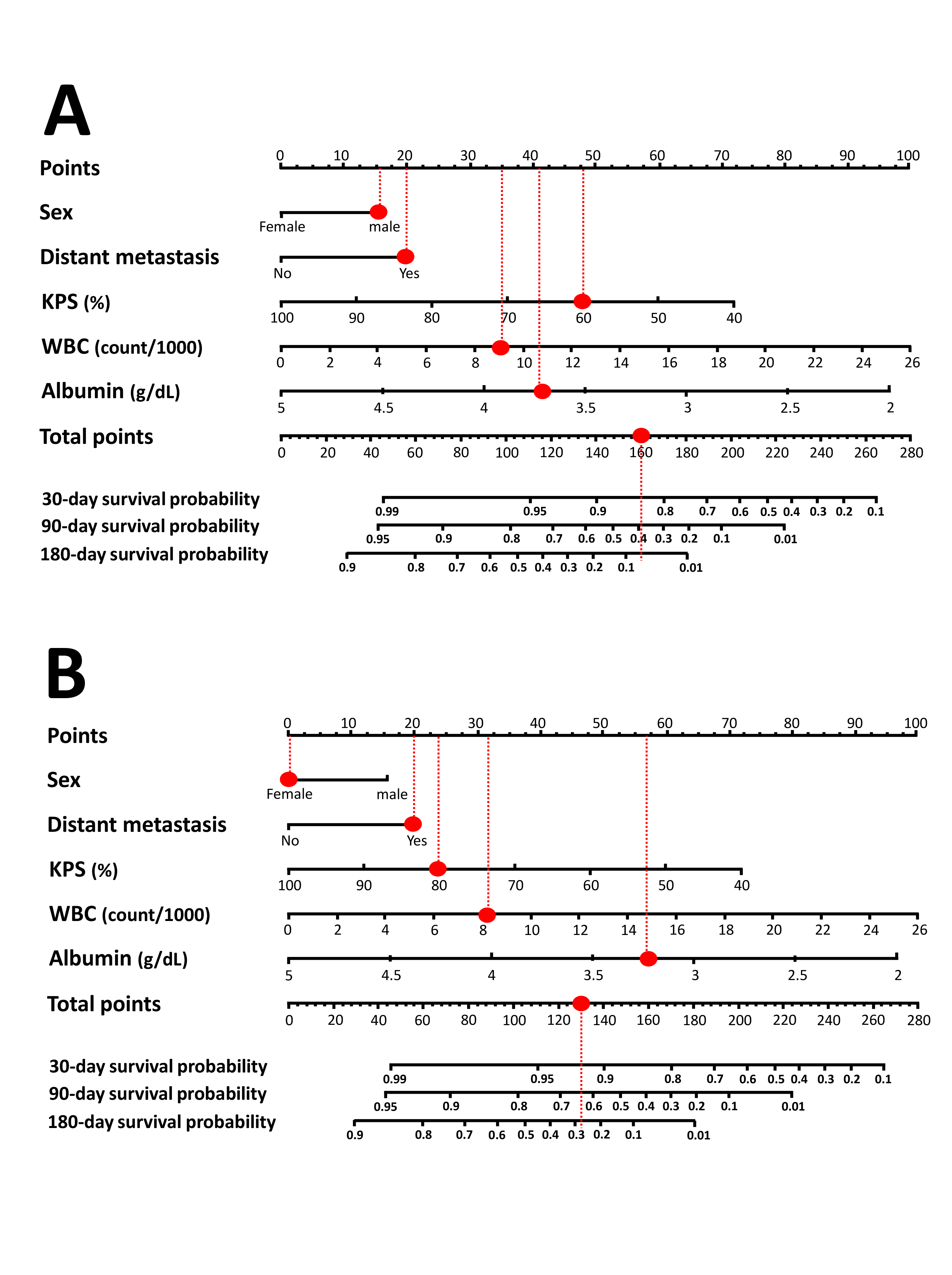

Supplement: Supplementary file 1 [file CAM4-7-3003-s001.tif]
