# Supplementary material for: Development and validation of a prognostic nomogram for ambulatory patients with advanced cancer
Source: Cancer Med. 2018 Jun 1;7(7):3003–10. doi: 10.1002/cam4.1582 (PMC6051167; doi:10.1002/cam4.1582)
Supplement: Supplementary file 2 [file CAM4-7-3003-s002.doc]

**Table S1.** Univariate Cox proportional hazards regression model (developmental sample)

| **Variable** | **Exp (B)** | **95% CI** | **P-value** |
| --- | --- | --- | --- |
| Age (per year) | 0.995 | 0.982-1.008 | 0.468 |
| Gender (female=yes) | 0.783 | 0.571-1.075 | 0.131 |
| Primary cancer type |  |  |  |
| Lung | 1.000 (Ref.) |  | 0.409 |
| Breast | 0.620 | 0.340-1.129 | 0.118 |
| Gynecological | 0.889 | 0.470-1.684 | 0.719 |
| UGI | 1.023 | 0.589-1.777 | 0.935 |
| LGI | 1.182 | 0.648-2.155 | 0.585 |
| Urological | 0.903 | 0.500-1.632 | 0.735 |
| Hematological | 0.231 | 0.069-1.236 | 0.094 |
| Skin and soft tissue | 1.074 | 0.480-2.403 | 0.862 |
| Head and neck | 0.628 | 0.318-1.244 | 0.182 |
| Unknown primary | 0.857 | 0.296-2.479 | 0.776 |
| Site of metastasis |  |  |  |
| Lung metastasis (yes) | 1.384 | 0.982-1.950 | 0.063 |
| Liver metastasis (yes) | 1.846 | 1.257-2.713 | 0.002 |
| Bone metastasis (yes) | 1.164 | 0.821-1.651 | 0.394 |
| CNS metastasis (yes) | 2.248 | 1.270-3.977 | 0.005 |
| Any metastasis (yes) | 1.690 | 1.193-2.395 | 0.003 |
| Palliative care only (yes) | 1.606 | 1.166-2.213 | 0.004 |
| KPS (per 10%) | 0.968 | 0.957-0.978 | <0.001 |
| BMI | 0.977 | 0.951-1.005 | 0.102 |
| Nasoenteric feeding tube | 1.160 | 0.569-2.365 | 0.683 |
| Gastrostomy feeding tube | 0.684 | 0.096-4.892 | 0.705 |
| Jejunostomy feeding tube | 1.755 | 0.947-3.252 | 0.074 |
| Ascitis | 1.245 | 0.740-2.096 | 0.409 |
| Peripheral edema | 1.511 | 0.952-2.398 | 0.080 |
| Hemoglobin, g/dL | 0.839 | 0.765-0.920 | <0.001 |
| WBC count, No. × 10^3^/µL | 1.000 | 1.000-1.000 | <0.0001 |
| Lymphocytes, No. × 10^3^/µL | 1.000 | 1.000-1.000 | 0.610 |
| Monocytes, No. × 10^3^/µL | 1.000 | 1.000-1.000 | 0.008 |
| Platelets, No. × 10^3^/µL | 1.002 | 1.001-1.003 | <0.001 |
| Calcium, mg/dL | 0.897 | 0.756-1.064 | 0.211 |
| LDH, U × 10^3^/L | 1.000 | 1.000-1.000 | <0.001 |
| Albumin, g/dL | 0.350 | 0.268-0.456 | <0.001 |
| CRP, mg/dL | 1.075 | 1.053-1.097 | <0.001 |
| Symptoms (ESAS) |  |  |  |
| Pain | 1.057 | 1.009-1.107 | 0.020 |
| Fatigue | 1.022 | 0.970-1.076 | 0.415 |
| Nausea | 0.979 | 0.916-1.046 | 0.527 |
| Depression | 0.963 | 0.910-1.019 | 0.195 |
| Appetite | 1.023 | 0.979-1069 | 0.306 |
| Dyspnea | 1.035 | 0.969-1.105 | 0.311 |
| EORTC QLQ-C30 |  |  |  |
| Global health | 0.992 | 0.986-0.999 | 0.030 |
| Physical functioning | 0.989 | 0.983-0.995 | <0.001 |
| Role functioning | 0.993 | 0.989-0.998 | 0.002 |
| Fatigue | 1.009 | 1.003-1.014 | 0.003 |
| Pain | 1.006 | 1.001-1.010 | 0.012 |
| Nausea/vomiting | 1.006 | 1.001-1.012 | 0.022 |
| Dyspnea | 1.004 | 0.999-1.009 | 0.136 |
| Appetite | 1.005 | 1.001-1.009 | 0.009 |

Legend: CI=confidence interval; Ref.=reference; UGI=upper gastrointestinal; LGI=lower gastrointestinal; CNS=central nervous system; KPS=Karnofsky Performance Status; BMI=body mass index; WBC=white blood cell; LDH=lactate dehydrogenase; CPR=c-reactive protein; ESAS=Edmonton Symptom Assessment System; EORTC QLQ-C30=European Organization for Research and Treatment of Cancer 30-item questionnaire.
